# Advances in natural product anti-coronavirus research (2002-2022)

**DOI:** 10.1186/s13020-023-00715-x

**Published:** 2023-02-13

**Authors:** Jiaxin Chen, Zuoqi Ding

**Affiliations:** 1 School of International Pharmaceutical Business, China Pharmaceutical University, Nanjing, Jiangsu China; 2 Editorial Board of Chinese Journal of Natural Medicines, Nanjing, Jiangsu China

**Keywords:** Coronavirus, Natural medicines, Traditional Chinese medicine, Coronavirus pneumonia

## Abstract

COVID-19 is a severe acute respiratory syndrome caused by coronavirus that has triggered acute respiratory infections in countries around the world. In the last 20 years, there have been several outbreaks of coronaviruses, which have had a tremendous impact on productive life and globalization. Since coronaviruses are mutagenic and highly susceptible to mutation, there are no specific drugs against coronaviruses. Medicines made from natural products gains worldwide attention, and the mechanism and effectiveness of natural products for the treatment of coronavirus-related diseases have received much attention after the global pandemic of COVID-19 in 2020. The vitro research results and clinical data from various countries have shown protective effects of good effects against coronaviruses. This review summarizes representative natural products for the treatment of coronavirus-related diseases in the past 20 years, and demonstrates the promising prospects of natural products against coronavirus-related diseases by listing herbal formulas, Chinese patent medicines and natural small molecule compounds and their therapeutic mechanisms, providing references for subsequent related studies.

## Background

At the end of 2019, a suddenly outbreak of pneumonia of unknown origin swept the world in a short period of time. The pathogen of this “unexplained viral pneumonia” was tentatively identified as Novel Coronavirus (Coronavirus Disease 2019, COVID-19) [1]. Since the beginning of the pandemic, countries around the world have been affected by novel coronavirus pneumonia to varying degrees, causing great damage to global economic production and livelihood. On January 30th, 2020, the WHO officially declared Novel Coronavirus pneumonia caused by COVID-19 as a global public health emergency [2]. Currently, COVID-19 continues affecting all aspects of our life, with sporadic outbreaks occurring in mainland China and more than 20,000 new confirmed cases per day in countries such as Japan, the United States, Switzerland and Russia. According to the latest WHO real-time statistics, as of 16:59 CET on October 4th (22:59 BST on October 4th), there had been 615,777,700 confirmed cases of NCCP and 6,527,192 cumulative deaths worldwide. 171,391 new cases of NCCP and 662 new deaths were reported on October 4th [3].

Coronaviruses are a group of viruses which is widely found in nature. They were first isolated from chickens in 1937, and in 1965, British scientists Tyrrell and Almeida et al. isolated the viruses from the nasal washings of a boy suffering from a cold, which was the first isolation of the viruses from humans, named at the time strain B814. These viruses were named coronaviruses since they were observed to be surrounded by bumps that resembled crowns [4]. Coronavirus is an RNA virus containing a huge genome, ranging from 15 to 27 kb in length for different viruses. It can affect the respiratory and gastrointestinal tracts of vertebrates by infecting them, so that the infections of coronaviruses are often accompanied by respiratory and digestive symptoms, and chronic infections can even affect the central nervous system [5]. In the last 20 years, there have been several outbreaks of acute respiratory infections caused by coronaviruses. In 2003, an outbreak of severe acute respiratory syndrome (SARS) caused by a novel coronavirus occurred in Guangdong Province, China. In 2012, a respiratory systemic disease caused by a SARS-like virus was identified in Saudi Arabia (Middle East Respiratory Syndrome, MERS). In late 2019, a new coronavirus pneumonia ravaged all over the world.

No specific drugs have been developed for the treatment of respiratory diseases caused by coronaviruses, and the vitro experiments and clinical trials on the diseases are still ongoing. Chloroquine derivatives are one of the experimental drugs in the anti-coronavirus pandemic [6], but follow-up studies shows no evidence of fast-going viral clearance in critically ill patients with COVID-19 treated with the combination of hydroxychloroquine and azithromycin [7]. Proteasome inhibitors such as nelfinavir and nirmatrevir exert antiviral effects by inhibiting viral replication [8, 9], but subsequent studies on nematavir have shown that nematavir has a significant effect in older patients and an insignificant effect on the rate of severe disease and mortality in younger patients [2]. The 2003 SARS epidemic also resulted in severe sequelae in cured patients due to hormone abuse. In the treatment of novel coronavirus pneumonia, 85% of patients received herbal medicine, and herbal medicine as an adjunct to COVID-19 treatment can effectively reduce the adverse effects produced by other conservative treatments [10].

**Fig. 1:**
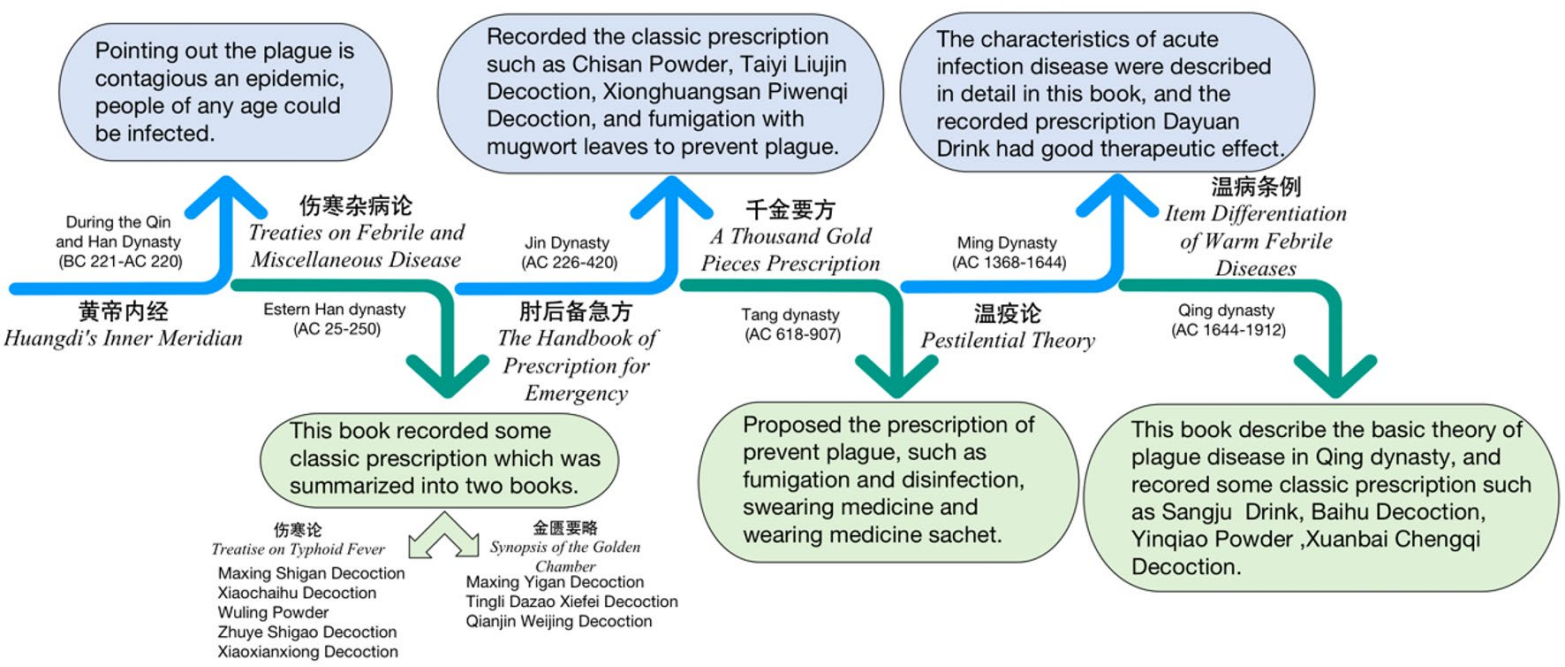
Plague and related treatment protocols were recorded in Chinese medical books of various dynasties, as early as hundreds of years ago, the Chinese people had a deep understanding of epidemics

Natural products are constituents or metabolic components of animals, plants, insects, marine organisms, or microorganisms, and include endogenous compounds in humans or animals. Natural products have anti-coronavirus effects [11]: Anthraquinone natural products, such as rhubarb acid, have anti-inflammatory effects; terpene natural products such as glycyrrhetinic acid can inhibit ACE2 binding to SARS-CoV-2 and achieve antiviral effects; flavonoid natural products such as baicalin can significantly inhibit coronavirus activity by inhibiting the 3CLpro protein in SARS-CoV-2; polyphenolic natural product, such as polydatin, has achieved anti-SARS-CoV-2 effect by binding ACE2 and inhibiting SARS-CoV-2 major protease (MPro). Most herbal medicines are natural products or derived from natural products. After being collected, processed and prepared, they will be used for Chinese medicine. Chinese medicine has a long history of treating plague. The earliest Chinese medical book, *Huangdi’s Inner Meridian*, considered plague as a “contagious and epidemic” disease, anyone, regardless age or gender, is at risk of infection. During the Eastern Han Dynasty, Zhang Zhongjing’s medical masterpiece, *Treatise on Febrile and Miscellaneous Diseases*, showed that the ancients had a specific understanding of infectious diseases at the time. Later generation summarized the book into *Treatise on Typhoid Fever* and *Synopsis of the Golden Chamber*, The classic formulas for the treatment of pestilence, such as Maxing Shigan Decoction, Xiaochaihu Decoction, Wuling Powder, Zhuye Shigao Decoction, and Xiaoxianxiong Decoction, were recorded in the *Treatise on Typhoid Fever*, and the formulas recorded in the *Synopsis of the Golden Chamber*, such as Maxing Yigan Decoction, Tingli Dazao Xiefei Decoction, and Qianjin Weijing Decoction, are also used to this day. *The Handbook of Prescription for Emergency* specifically compiled in the Jin dynasty, listed the prescriptions for the treatment and prevention of pestilence at that time, such as Chisan Powder, Xionghuangsan Piwenqi Decoction, Taiyi Liujin Decoction. this book also told people to use mugwort fumigation to prevent plague. In the Tang dynasty, *the Treatise on Typhoid Fever* was summarized and organized in Sun Simiao's book, *A Thousand Gold Pieces Prescription,* in which the chapter of “Prevention of Warmth” recorded the prescriptions for the treatment of pestilence and proposed the methods of fumigation and disinfection, swearing drug, and wearing medicine sachet. *Pestilential Theory*, written by Wu Yoke in Ming dynasty, is an epoch-making work describing acute infectious diseases and is an important manifestation of the original theory and clinical practice of Chinese medicine. Wu Youke pioneered the Dayuan drink for the treatment of epidemic disease, with the good effect. In the Qing dynasty, Wu Huang wrote *Item Differentiation of Warm Febrile Diseases* in which the classical prescriptions for pandemic disease, such as Sangho Drink, Baihu Decoction, Yinqiao Powder and Xuanbai Chengqi Decoction were recorded, forming the basis of pandemic disease theory in the Qing dynasty. The records related to plague in ancient Chinese medical books are shown in Fig. 1. With the development in the past thousands of years, Chinese medicine has accumulated a rich theoretical basis for the treatment of infectious diseases caused by coronavirus, which is a natural advantage of China, showing the great potential of Chinese medicine in the development of clinical practice against coronavirus infection.

### Anti-coronavirus effect of natural products

Natural products have made significant contributions to human health for thousands of years, and China has been very concerned about the active ingredients of natural products, and Professor Tu Youyou won the 2015 Nobel Prize in Physiology and Medicine for the discovery of artemisinin, the active product in *Artemisia annua* for the treatment of malaria. Natural products usually exhibit a wide range of biopharmacological effects through multiple pathways and targets, with mild adverse effects and relatively low drug resistance, making them suitable for long-term use [2]. Many natural products have significant therapeutic effects, for example, the active substance in *Ginkgo* is quite popular in the German market, and Germany imports a large amount of *Ginkgo* leaves from China every year to prepare related formulations [12]. Calanolide A is a coumarin compound isolated from Malaysian *Photinia serrulate* with potent anti-HIV activity, of which related drug has entered phase II clinical trials, showing vast application prospects [13]. In the United States, one-third of FDA-approved marketed drugs are natural products [14]. Since outbreak of COVID-19, scholars around the world have renewed their interest in the active ingredients of natural drugs.

**Fig. 2:**
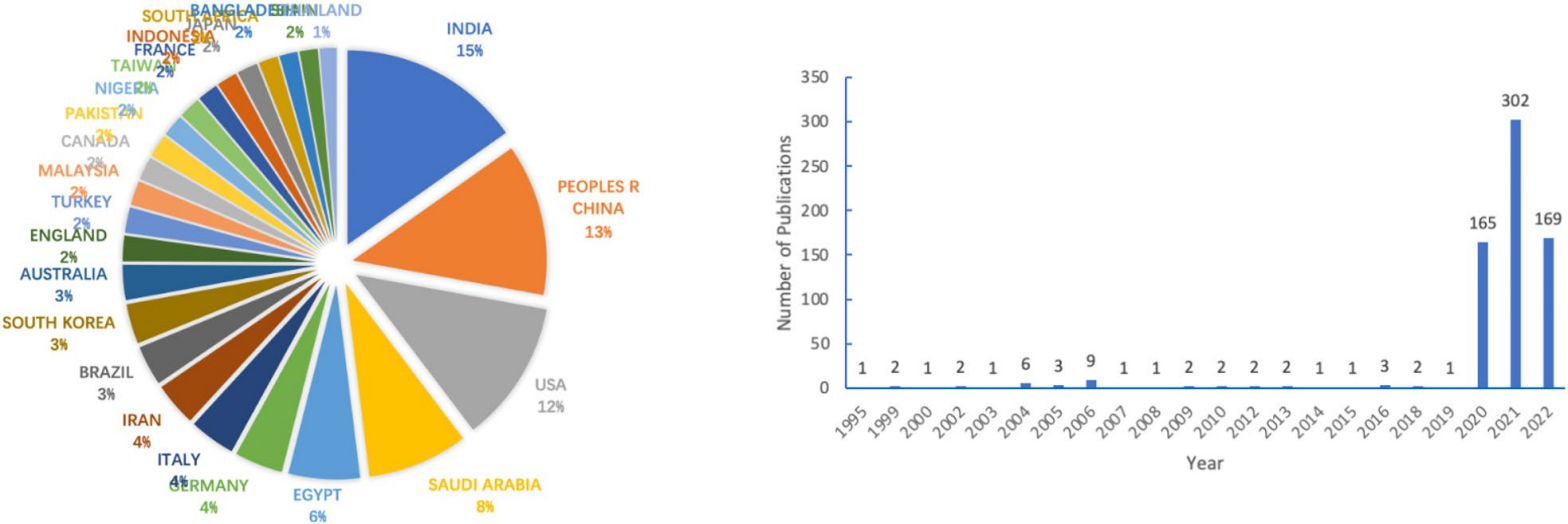
Article production and author distribution of natural products against coronavirus. The figure shows that the number of articles on natural products against COVID-19 has increased significantly from 2020, with the majority of authors coming from India, China and the USA

Figure 2 shows the distribution of authors studying coronaviruses and natural products by country and region(the date from Web of Science: Analyze results: natural product (Topic) and COVID-19 (Topic)) [15], and demonstrates the number of studies related to natural drugs and coronavirus published over the years related, from which India scientists are more concerned about the topic, followed by China and the USA. Additionally, the figure showing the increased enthusiasm of scholars in different countries for research on natural drugs for coronaviruses in 2003 after the SARS epidemic. After the outbreak of COVID-19 in late 2019, there was an explosive increase in research output on natural products for coronavirus-related diseases.

Many ancient Chinese texts have recorded formulas treating epidemic, some of which are even used today. For example, the Qingfei Paidu Decoction currently used in clinical practice to treat patients with COVID -19 is the innovative combination of five classical Chinese medical formulas, including Maxing Shigan Decoction, Shegan Mahuang Decoction, Xiaochaihu Decoction, Wuling Power, and Ganju Decoction. Clinical evidence from the fight against SARS shows that the combined treatment of Chinese and Western medicine can alleviate the symptoms of SARS patients, improve the quality of life and accelerate the absorption of pulmonary infiltrates [16], while reducing the dosage of glucocorticoids and the side effects caused by hormones. Xiaochaihu Decoction and others are also widely used in febrile diseases such as influenza A and pneumonia [17]. Chinese medicinal preparations also play an important role in the fight against COVID-19, and t*he Clinical Guidelines for the Treatment of Novel Coronavirus (2019-nCoV) Pneumonia (Trial Version 7)* recommends the clinical use of Huayu Huadu Baishi Formula for the treatment of the symptoms caused by COVID-19, such as cough, blood in sputum, irregular bowel movements and red tongue [18]. On the May 4th, 2020, the State Drug Administration issued the Supplementary Application for Drug Approval for the Chinese patent medicines Xuebijing Injection and Lianhua Qingwen Capsule officially identifying them for the treatment of novel coronavirus pneumonia [19]. In addition, it has also been shown that disease progression in COVID-19 patients is associated with hematological and immunological responses, and the active ingredients of Chinese medicine may counteract COVID-19 in both aspects, in which the potential mechanisms are still under study [20].

### Mechanism of action of natural products against coronavirus-related diseases

Currently, the main mechanism considered for the pathogenesis of COVID-19 is that SARS-CoV-2 infection triggers an imbalance in the immune regulation of the body, which generates cytokine storm and an excessive immune response stress and causes apoptosis, ultimately leads organ damage [21]. The active ingredients of natural products have the function of treating the disease and enhancing hosts immunity for the treatment and prevention of COVID-19 in 3 main aspects: antiviral activity, anti-inflammatory activity and modulation of the immune pathway.

### Antiviral treatment

**Fig. 3:**
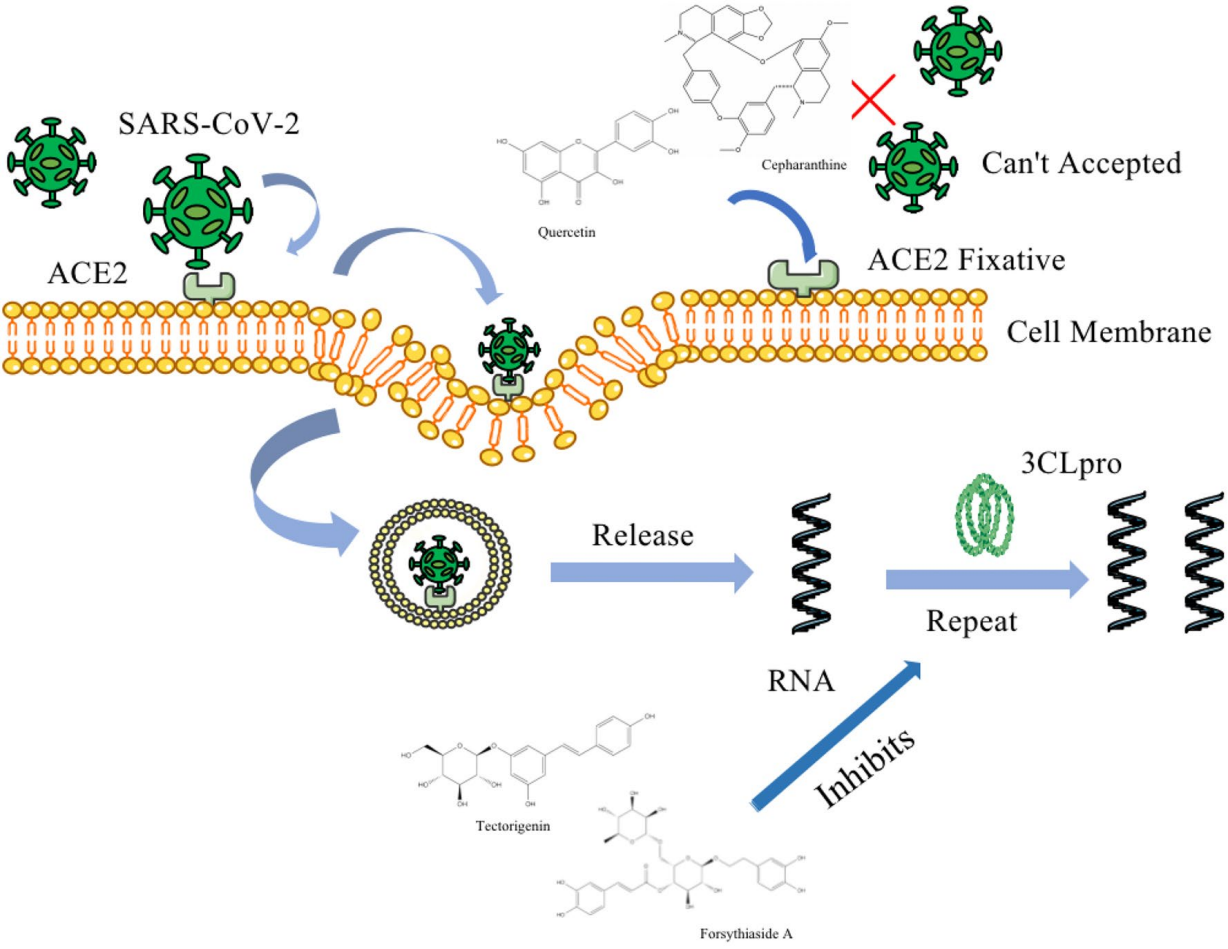
The process of viral invasion into the cells of the body, natural products block coronavirus invasion by occupying ACE2 receptors and inhibits viral replication via 3CL pro

Coronavirus main protease 3CLpro is a specific cysteine protease in the coronavirus family. Being a key enzyme for viral replication, it plays an important role in the life cycle of coronaviruses and is highly conserved, making it an important target for broad-spectrum antivirals [2]. Flavonoids, terpenoids, and polyphenols in extracts of *Scutellarin baicalensis, Forsythia lanceolata, Radix Rehmanniae*, and *Radix tigrinus* can achieve anti-viral effects by inhibiting the activity of 3CLpro [23, 24]. Angiotensin-converting enzyme II (ACE2), a receptor for SARS-CoV-2 entry into host cells, is widely distributed in the human body. It is involved in the invasion of coronavirus into cells [25], and the antiviral effect of natural drugs can also be achieved in seaweed extract by immobilizing the ACE2 receptor to block the binding of the virus to the ACE2 receptor of the host [26]. Figure 3 illustrates the process of viral invasion into the cells of the body using the example of cepharanthine.

### Anti-inflammatory treatment

Cytokine storm is one of the main mechanisms of coronavirus pathogenesis. Cytokines are involved in cell growth, differentiation, apoptosis, inflammatory host defense, and restoration of homeostasis [27]. Cytokine storm is a phenomenon in which infection of the organism with microorganisms triggers rapid and massive production of multiple cytokines in body fluids, so that mild symptoms can lead to multiple organ dysfunction syndrome (MODS) and even lead the patient dead. Therefore, upon SARS-CoV-2 invasion, the organism immediately produces inflammatory responses, leading to cytokine storm. The active components of natural products can inhibit inflammation through multiple pathways at various targets and different levels to reduce the rate of severe cases and mortality, exhibiting great potential in the treatment of inflammation and inflammation-related diseases [28]. For example, a significant reduction in cytokine levels was found after feverfew injections to rats in lung injury model [10]. Carthamin yellow A from the plant Saffron in the family *Asteraceae* had a significant anti-inflammatory effect in mice [19]. The NF-κB signaling pathway is also important in coronavirus pathogenesis. NF-κB is a nuclear transcription factor in cells that specifically binds to the upstream enhancing self-sequence of immunoglobulin κ light chain genes and activates gene transcription. NF-κB signaling pathway regulates transcriptional processes involved in a variety of inflammatory responses (e.g., IL-1, IL-6, TNF-α), adhesion factors and protease-like genes in response to a variety of extracellular signaling stimuli, generating immune, inflammatory and stress responses and affecting cell proliferation. Flavonoids such as baicalin can achieve inflammation control by inhibiting NF-κB activation and IκB degradation [29, 30].

Innate immune dysregulation is an important feature of severe COVID-19 [31]. Dendritic cell (DC) play an important role in the innate immune response against viral infection. When activated, DCs move into lymphoid tissues to interact with T and B cells to stimulate control of the immune response. DC senses pathogens through TLR7 and produces high levels of pro-inflammatory factors in response, leading to a large number of inflammatory factors, including INF-α, TNF-α, IL-6 [32, 33]. Several studies have shown that natural products can modulate the phenotype and function of DC and have been shown to be effective in clinical applications. Resveratrol exerts immunosuppressive effects by down-regulating DC differentiation and maturation and inhibiting T-cell activation [34]. Luteolatine can block the NF-κB signaling pathway induced by lipopolysaccharide (LPS) and inhibit the expression of pro-inflammatory genes in DC [35]. Astragalus extract can inhibit LPS induced secretion of inflammatory factors in DC to achieve anti-inflammatory effects [36, 37].

**Fig. 4:**
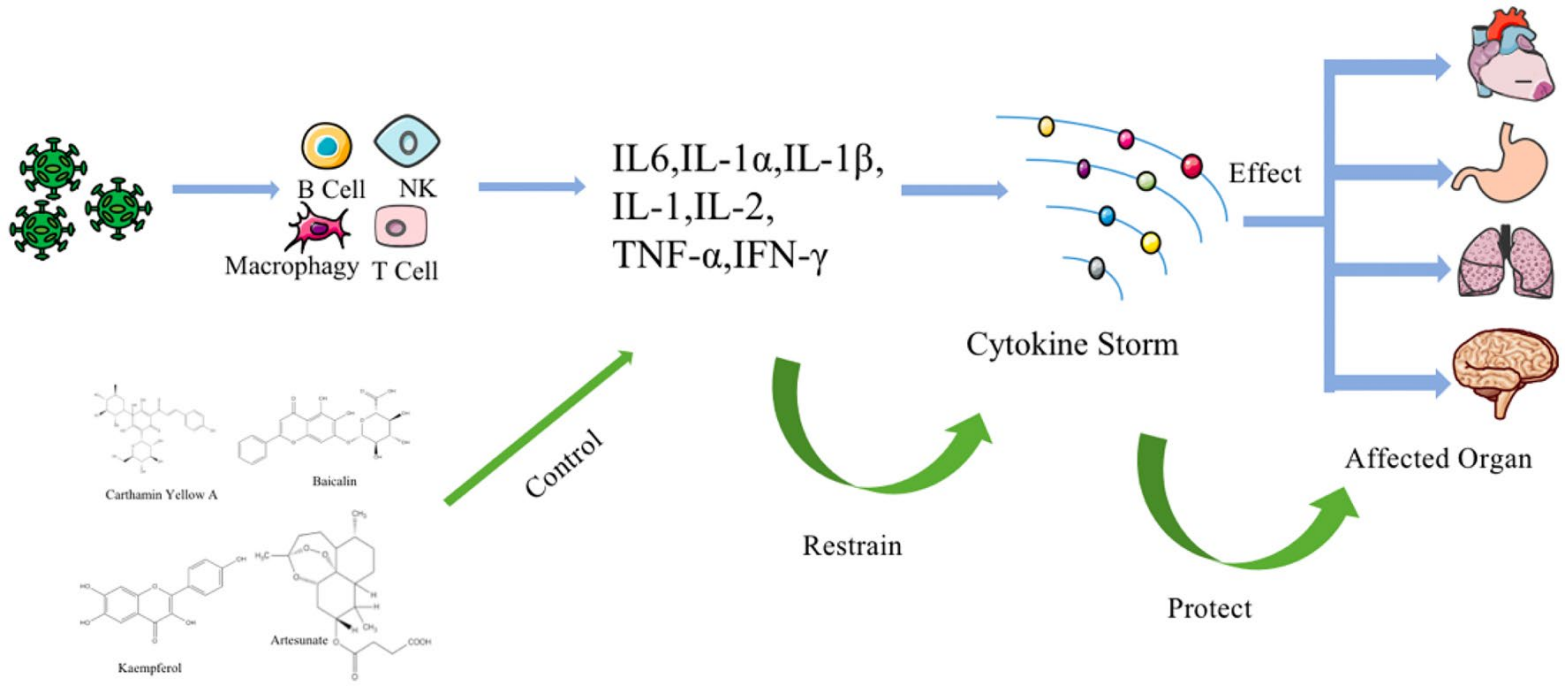
The anti-inflammatory mechanism of natural products against cytokine storm. Natural products protect the organism by inhibiting the cytokine storm triggered by coronavirus, which can affect organs

**Fig. 5:**
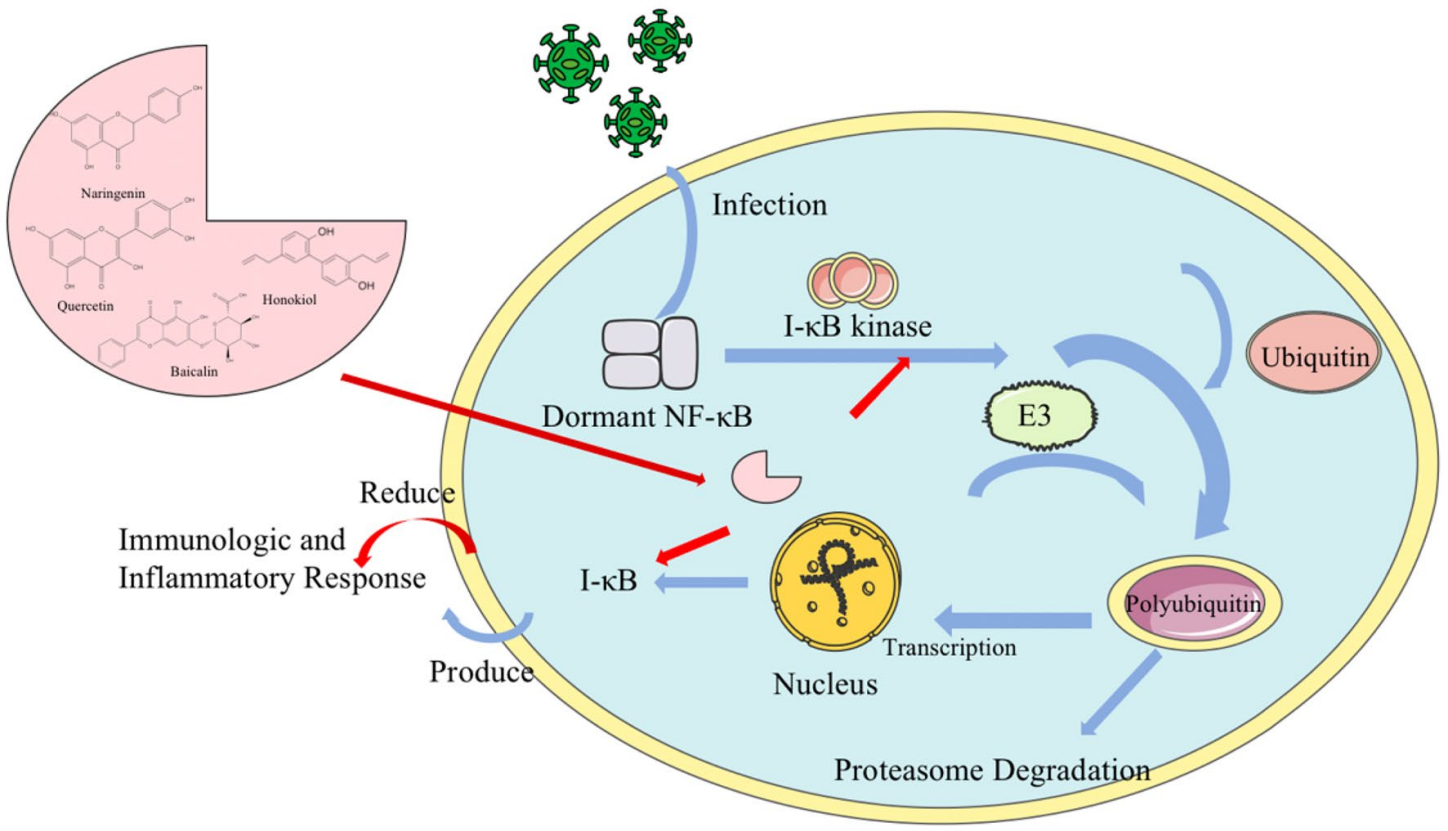
The mechanism of natural products inhibiting inflammation through NF-κB signaling pathway. Natural products control inflammation by inhibiting NF-κB activation and IκB degradation

Figure 4 shows the anti-inflammatory mechanism of natural products against cytokine storm. Figure 5 shows the mechanism of natural products inhibiting inflammation through NF-κB signaling pathway.

### Modulation of immunity

Natural products also have the function of regulating immune imbalance [10]. T cell receptor (TCR) pathway is a pathway related to immune regulation. T cells can effectively clear pathogens that invade the body and infect cells, and it plays a key role in the adaptive immune response against infection [38]. The active ingredients in Sangju Drink, Yupingfeng Powder and Qianjin Weijing Decoction can stimulate T-cell induction and improve immunity. It also been suggested by that natural products with anti-coronavirus activities are the main ingredients of some common dietary supplements that can be used to boost immunity in the general population during epidemics [38].

### Anti-coronavirus prescriptions and proprietary Chinese medicines

Chinese people has known about plague since two thousand years ago. The nature of plague has been described in detail in *Huangdi's Inner Meridian* written during the Qin and Han dynasties, and many classical prescriptions for the treatment of epidemics have been recorded in the *Treatise on Febrile and Miscellaneous Diseases* written by Zhang Zhongjing, a famous doctor in the Eastern Han dynasty. After the outbreak of the COVID-19, the “Three Medicines and Three Formula” were recommended in the “Novel Coronavirus Pneumonia Treatment Plan” promulgated by the National Health Commission, which are clinically screened formulas for the effective treatment of COVID-19 [40]. Most of the formulas are based on the innovation of formulas recorded in ancient medical books. The formula of Qingfei Paidu Decoction is based on the combination of Maxing Shigan Decoction, Shengan Mahuang Decoction, Xiaochaihu Decoction, Wuling Powder, and Juzhi Decoction, which have played an important role in blocking the progression of the disease, improving the symptoms and shortening the course of the disease. Huashi Baidu Formula is a combination of Maxing Shigan Decoction, Tingli Dazao Xiefei Decoction, Xuanbai Chengqi Decoction, and Huopu Xialing Decoction, which has antiviral effect and enhances immunity. The Xuanfei Baidu Formula is suitable for mild cases and can shorten the duration of infection of patients' symptoms. Yinqiao Powder is commonly used in the treatment of influenza and clinical trials on the efficacy and safety of Yinqiao Powder plus or minus formula in the treatment of COVID-19 are underway (ChiCTR2200066185). Jinhua Qing Gan Granule used in influenza A H1N1 is the combined formula of Yinqiao Powder and Maxing Shigan Decoction modified with addition and reduction [41], which is the first evidence based proprietary medicine that has undergone phase III clinical trial in the history of Chinese medicine [42]. In addition, Professor Zhang Boli and his team developed the “COVID-19 No. 2 Formula” based on Tingli Dazao Xiefei Decoction and Maxing Shigan Decoction for ordinary cases of COVID-19 [43], which can effectively prevent the progression of the disease. Hunan Province has recommended “Prevention No. 1” and “Prevention No. 2”, which can regulate the immune function of weak people and effectively prevent COVID-19 infection [11, 43]. On March 21st, 2021, the State Drug Administration approved the marketing of Qingfei Paidu Granule, Huashi Baidu Granule and Xuanfei Baidu Granule, which are derived from ancient Chinese classical prescriptions [44].

There are also some formulas that are not yet used clinically for the treatment of COVID-19, but they have shown improvement in the symptoms of respiratory and pulmonary diseases, and it can be speculated that these formulas have potential for the treatment of COVID-19. Table 1 lists some classical formulas from ancient Chinese medical texts, providing references for subsequent clinical applications in the treatment of coronavirus-related diseases.

**Table 1:** Classical formulas from ancient Chinese medical texts

Name of formula	Main component drugs	Main mechanisms	References
Yupingfeng Powder	Astragalus Membranaceus, Atractylodis Macrocephalae Rhizoma	Reduces TNF- α and IL-6 levels, inhibits phosphorylation and chemotaxis of JAK1/STAT3 pathway, thereby reducing IL-8 production, and regulates MMP-9/TIMP1 homeostasis, thereby inhibiting airway inflammatory response and airway remodeling	[10, 45]
Maxing Shigan Decoction	Ephedra Herba, Armeniacae Semen, Gypsum Fibrosum, Glycyrrhizae Radix	Reduces the inflammatory response and modulates the immune system by regulating several proteins that interact with ACE2 and several signaling pathways associated with disease development	[46]
Xiaochaihu Decoction	Bupleuri Radix, Scutellariae Radix, Pinelliae Rhizoma, Glycyrrhizae Radix	Immobilizes ACE2 and prevents the virus from binding to ACE2 to achieve antiviral effect	[17, 47]
Yinqiao Powder	Lonicera Japonica, Fructus Forsythiae, Platycodon Grandiflorum	Exerts anti-inflammatory effect through mediating NF-κB signaling pathway, B-cell signaling pathway and T-cell signaling pathway	[48]
Sangju Drink	Folium Mori, Flos Chrysanthemi, Armeniacae Semen	Exerts anti-inflammatory effect through mediating NF-κB signaling pathway, B-cell signaling pathway and T-cell signaling pathway	[48]
Dayuan Drink	Areca Catechu, Magnolia Officinalis, Amomum Tsao-ko	Acts on PIK3CG, AKT1, IL-4, IL-6, IL-7 and other targets to reduce oxidative stress and achieve anti-inflammatory and immunomodulatory effects	[27]
Xuanbai Chengqi Decoction	Gypsum Fibrosum, Rheum Officinale, Armeniacae Semen	Reduces TNF-α and IL-1β levels and improves IL-10, thereby reducing the inflammatory response	[17]
Maxing Yigan Decoction	Ephedra, Armeniacae Semen, Pearl Barley, Glycyrrhizae Radix	Influences AGE-RAGE, IL-17, tumor necrosis factor (TNF) and other signaling pathways through IL-6 to achieve anti-inflammatory and anti-viral effects	[49]
Tingli Dazao Xiefei Decoction	Draba Nemorosa, Fructus Ziziphi Jujubae	Decreases serum levels of IL-6 and TGF-β1, increases IL-10 levels, suppresses inflammatory responses and improves histopathological changes in the lung	[50]
Qianjin Weijing Decoction	Rhizoma Phragmitis, Pearl Barley, Seed of Chinese Waxgourd, Peach Kernel	Reduces the level of inflammatory factors such as IL-6, inhibits the release of inflammatory factors from helper T cells, and promotes the release of anti-inflammatory factors from regulatory T cells to achieve anti-inflammatory effects	[17]
Prevention of “COVID-19” No. 2 formula	Astragalus Membranaceus, Lonicera Japonica, Citri Pericarpium, *Fructus Ziziphi Jujubae, Glycyrrhizae Radix*	Exert immunomodulatory effects on CsA-induced immunodeficiency mouse model by improving thymic tissue structure and regulating the expression levels of IFN-γ and Ang II	[43]

In addition to classical formulas, the National Health Commission has approved several proprietary Chinese medicines in the past 20 years, which have played a vital role during epidemics or when the stockpile of potent drugs is insufficient. Both Lianhua Qingwen Capsule and Xuebijing Injection were approved and marketed during SARS in 2003. Lianhua Qingwen Capsule can significantly inhibit SARS-CoV-2 replication and affect virus morphology in vitro [51], while significantly inhibiting the overexpression of inflammatory factors TNF-a, IL-6, MCP-1 and IP-10 caused by SARS-CoV-2 and exerting anti-inflammatory activity [52]. Lianhua Qingwen Capsule has been included in the new coronavirus treatment protocols in several countries and regions due to its significant effect [53], and has entered phase II clinical trials in the United States [39]. Xuebijing Injection is derived from Xuefu Zhuyu Decoction, which is a famous formula in Qing dynasty. It can treat infection-induced systemic inflammatory response syndrome and multiple organ dysfunction syndrome. It is a major achievement in the prevention and treatment of serious infectious diseases and fills the gap in the treatment of sepsis and multi-organ dysfunction syndrome [42]. Xuebijing injection has a good effect on the treatment of severe COVID-19 patients. It can shorten the mechanical ventilation time and antibiotic use time of severe COVID-19 patients and improve inflammation indicators [54]. A multi-center prospective study of Xuebijing Injection in the treatment of severe novel coronavirus pneumonia by Academician Zhong Nanshan's team evaluated the improvement effect of Xuebijing injection on the pneumonia severity index (PSI) and prognostic influence. The research results showed that conventional treatment combined with Xuebijing injection could significantly improve the PSI risk rating and clinical prognosis but did not increase the drug safety risk [55]. Zhang et al. conducted a multicenter prospective cohort study of Jinhua Qinggan Granules treating adult patients with COVID-19, and observed that the cough recovery rate in the treatment group was significantly higher than that in the control group, and the duration of the virus was shortened [56]. During the influenza A H1N1 in 2009, the State Drug Administration approved the listing of Jinhua Qinggan Granule, which showed comparable efficacy to Tamiflu with no side effects in evidence-based research [42]. On April 5th, 2020, the State Drug Administration issued the “Supplementary Application for Drug Approval”, in which the Chinese patent medicines Xuebijing Injection and Lianhua Qingwen Capsule were officially determined to be available for the treatment of novel coronavirus pneumonia [17].

During the COVID-19 epidemic, both national and local health committees were recommended for the treatments of coronavirus pneumonia, such as “Pneumonia No. 1”, or in other word, Toujie Quwen Granule, which is a combination of Xiaochaihu Decoction, Xiaoxianxiong Decoction and Dayuan Drink with no adverse effects and good safety [57]. It has been clinically proved to be effective in reducing fever symptoms, controlling disease progression and reducing pneumonia complications [58, 59]. In addition, daily medications such as Shuanghuanglian Oral Liquid, Qingkailing Injection and Huoxiang Zhengqi Capsule can also be used to improve symptoms, and classical medicines such as Angong Niuhuang Pills also have potential to treat coronavirus disease.

For reference, Table 2 lists Chinese patent medicines or recommended prescriptions that can be used to treat or have the potential to treat coronavirus disease.

**Table 2:** Chinese patent medicines or recommended prescriptions that can be used to treat or have the potential to treat coronavirus disease

Name of proprietary Chinese medicine	Drug effect	Mechanism	References
Lianhua Qingwen Capsule	Relieves fever, cough, malaise and prevents disease progression	Inhibits replication of SARS-COV-2 virus, affects viral morphology, and produces anti-inflammatory effects in vitro	[51]
Jinhua Qinggan Granules	Exerts antipyretic and anti-inflammatory, and immunomodulatory effect	Regulates interleukin receptor and mitogen-activated protein kinase receptor, stimulates autoimmunity, inhibits RNA polymerase and thus inhibiting virus replication	[41]
Xuebijing Injection	Improve cure rates in critically ill patients	Inhibits viral replication and reduce virus-induced cell death and inflammatory response in vitro	[19, 60]
Qingwen Hufei Granule	Exerts anti-inflammatory and antipyretic effects, clears the lung and relieves cough	Prevents the virus from entering the host cell and binding to ACE2, prevents the virus from entering the host cell, blocks the SARS-CoV-2 virus protein from binding to SARS-CoV-2 3CL hydrolase, and blocks the process of viral RNA replication, thus exerting a control effect	[61]
Reduning Injection	Treats upper respiratory tract infections caused by high fever, slight evil wind and cold, head and body pain, cough, yellow sputum, etc	Reduces IL-1β, TNF-α, IL-4, IL-6, IL-8, IL-10, ICAM-1, NF-κB and controls cytokine storm	[62]
Toujie Quwen Granule (Pneumonia NO.1)	Improves patient symptoms, shortens the time to fever reduction, and reduces the incidence of severe pneumonia	Mediates multiple immune and inflammation-related pathways to achieve anti-inflammatory, anti-infective, and immune modulating effects	[57]
Qingkailing Injection	Exerts anti-inflammatory and antipyretic effect against upper respiratory tract infections	Regulates immune function, suppresses cytokine storms and reduces free radical buildup	[63]
Reyanning Granule	Relieves cough and phlegm, relieves fever and inflammation, and relieves upper respiratory tract symptoms	Inhibits auricular swelling in mice caused by xylene and foot and plantar swelling in rats caused by egg white	[64, 65]
Qingfei Dayuan Granule	Treats pneumonia through antipyretic, and anti-inflammatory effects and immune function regulation	Acts on NF-κB, TNF, MAPK3, IL-1β, PTGS, CASP3 and other targets to exert various pharmacological functions such as antipyretic, anti-inflammatory and immune modulation	[66]
Angong Niuhuang Pills	Treats of critically ill patients	Shows strong binding activity with ACE2 protein, prevents viral entry into host cells and has potential to treat coronavirus disease	[24]
Huoxiang Zhengqi Capsule	Improves weakness, and gastrointestinal symptoms	Modulates anti-inflammatory and immunological functions and intervenes airway remodeling to exert anti-coronavirus disease effects	[67, 68]
Shuanghuanglian Oral Liquid	Treats fever, cough, and sore throat	Inhibits the activity of 3CLpro and suppresses viral replication	[23]
Tanreqing Injection	Treatment of acute and chronic bronchitis, pneumonia and upper respiratory tract infections caused by bacteria or viruses	By acting on ACE2 receptors and inhibiting the activation of NF-κB in the body, it can achieve antiviral and anti-inflammatory effects, and produce therapeutic effects on infection, inflammation and lung injury caused by COVID-19	[69]

### Natural product extracts for anti-coronavirus

National and local health committees have recommended Chinese patent medicines or prescriptions for the prevention and treatment of COVID-19, many of which contain drugs such as *Lonicera Japonica, Astragalus membranaceus, Fructus Forsythiae* and *Polygonum Cuspidatum,* which were also common herbs in ancient prescriptions for the treatment of plague. It can be presumed that the extracts of these plants contain active ingredients against coronavirus. The active ingredients and the targets of action can be analyzed by using modern science and technology.

Some natural drug extracts can produce antiviral effects by interfering the assembly of the virus to the host cells. Anticoagulant heparin extracted from edible seaweed has a strong affinity to the stinging protein of SARS-CoV-2, and along with other related polysaccharides, it has shown antiviral properties in vitro [26]. The heparin variant that removes the anticoagulant personality can inhibit the binding of SARS-CoV-2 to the ACE2 receptor of the host, thereby blocking the viral infection. Tthe active component SSP of chicken blood vine can adhere to respiratory mucosa for a longer period and close the ACE2 receptor on the cells of the organism, thereby blocking the binding of the virus to the receptor on the cell membrane [70].

Some natural product extracts may have antiviral effects by inhibiting viral replication. 3-chymotrpsin-like protease (3CLpro) is a key enzyme in coronavirus replication. *Scutellaria baicalensis* extract has a good safety profile and is widely used. It’s ethanolic extract inhibited SARS-CoV-2 3CLpro activity and SARS-CoV-2 replication *in vero* cells. Zhang Boli added extracts of *Polygonum Cuspidatum* in Xuanfei Baidu Decoction, which has anti-inflammatory and antiviral activities. It’s active ingredients such as isoquercitrin can bind with tryptophan residues of MERS-CoV 3CLpro and inhibit the activity. Polygonin and resveratrol have high affinity with SARS-CoV-2 3CLpro, SARS-CoV 3CLpro and MERS-CoV 3CLpro, and are potential broad-spectrum inhibitors of coronavirus [71].

Lonicera japonica extract can reduce damage to organs by inhibiting cell storms. Some studies have shown that Lonicera japonica extract can reduce the degree of ALI injury in rats by inhibiting NF-KB signaling pathway [72]. At the same time, the TNF-α and IF-1β of pneumonia mice inoculated with influenza A adapted strain could be inhibited, effectively blocking the excess autoimmunity caused by influenza virus [73].

Some of the natural product extracts and their mechanisms of action are shown in Table 3.

**Table 3:** Sources of some natural product extracts and their mechanisms of action

Name of extracts	Source plant	Active sulfated polysaccharides	Effect	References
Seaweed Extract	Edible seaweed	Anticoagulant heparin	Blockade of ACE2 receptors from binding to proteins	[26]
*Spatholobus Suberectus* Dunn Extract	*Spatholobus Suberectus* Dunn	proanthocyanidins (PACs), flavonoids, Phenolic compounds	Blockade of spinosin receptors and host ACE2 receptors	[70]
*Polygonum Cuspidatum* Extract	*Polygonum Cuspidatum*	Isoquercitrin, Polydatin, Resveratrol	Inhibits the activity of SARS-CoV-2 3CLpro	[74]
Lonicera Japonica Extract	Lonicera Japonica	Flavonoids, Polysaccharides	Inhibite the NF-κB signaling pathway, and reduce inflammation by inhibiting the production of pro-inflammatory cytokines, chemokines, IL-1β, TNF-α, and IL-6. Promotion of Th1-type cell expression and enhances immunity	[21, 75]
Ethanolic extract of *Scutellaria baicalensis*	*Scutellaria baicalensis*	Baicalin and baicalein	Inhibited SARS-CoV-2 3CLpro activity and SARS-CoV-2 replication	[76]
*Salvia Miltiorrhiza* Extract	*Salvia Miltiorrhiza*	Tanshinones (dihydrotanshinones, etc.)	Inhibit TLR4/NF-κB mediated inflammatory response and play an anti-acute lung injury role and inhibit the activity of SARS-CoV 3CLpro and prevent virus replication	[71, 73]

### Representative natural molecule compounds against coronavirus

The previous section lists formulas and proprietary Chinese medicines for coronavirus disease, and some natural drugs appear several times in these formulas and proprietary Chinese medicines, such as *Fructus Forsythiae, Scutellaria baicalensis* and *Glycyrrhizae Radix*. In which molecule compounds such as baicalin, luteolin and quercetin are the active ingredients for the treatment of coronavirus. The targets of active natural compounds are mainly the inhibition of inflammatory pathways, the immobilization of ACE2 protein, and the inhibition of viral replication. Glycyrrhetinic acid, the main active ingredient of *Glycyrrhizae Radix*, which can inhibit viral replication and has been used in the treatment of malignant tumours, HIV-1 and HCV [77, 78]. Its derivative, diammonium glycyrrhizate, has good efficacy in acute lung injury. Intravenous administration of diammonium glycyrrhizate to patients with acute lung injury can inhibit the expression of TNF-α and promote the secretion of the anti-inflammatory factor IL-10 in vivo, thereby reducing the lung tissue damage caused by over-immunization [79]. Clinical trial of diammonium glycyrrhizate in combination with vitamin C for COVID-19 is ongoing (ChiCTR2000029768). Quercetin and kaempferol, the active ingredients in *Fructus Forsythiae*, *Scutellaria baicalensis*, have significant anti-inflammatory effects and can inhibit cytokine storm. Artemisinin and its derivatives also have the potential to treat COVID-19. Artemisinin has shown good efficacy in respiratory diseases, acute lung injury and the ability to reduce the expression of pro-inflammatory factors such as IL-6 in COVID-19 patients, thereby inhibiting the development of cytokine storms [78]. Interventional clinical trial of artemisinin-pipequine tablets for the treatment of patients with mild and common types of COVID-19 is ongoing (ChiCTR2000033049). Matrine also has anti-inflammatory effects. Hou et al. conducted a comparative study on the effects of compound Matrine injection and Xuebijing Injection on acute lung injury in rats, and the results showed that compound Matrine injection had more obvious effects on the treatment of acute lung injury and the inhibition of inflammation [80]. Its clinical effect is also relatively good, as for matrine sodium chloride injection in the treatment of 40 COVID-19 patients, the therapeutic efficiency was 100% [81].

Both domestic and international research teams have paid more attention to the anti-coronavirus effects of natural small molecule compounds. Liu et al. found that quercetin was the most effective ACE2 fixative among the polyphenolic compounds used in the experiments [82], which could effectively combat viral invasion of the organism. Ohashi et al. in Japan Drayman in America found that Cepharanthine had highest anti-coronavirus activity of any single drug analyzed, and even had better therapeutic potential than redesivir [83, 84]. PharmDrug, a Canadian pharmaceutical company, agreed with the FDA in November 2021 to use Cepharanthine for the treatment of patients with mild to moderate COVID-19, and will start a study in the second half of 2022 [84]. Liu et al. found that flavonoids in *Scutellaria baicalinas*e, such as baicalein, have strong activity in inhibiting viral replication [85]. Some natural molecule compounds with therapeutic effects or potential for the treatment of coronaviruses and their targets of action are listed in Table 4 for reference.

**Table 4:** Natural molecule compounds with therapeutic effects or potential for the treatment of coronaviruses and their targets of action

Structure and name	Source plant	Drug effect	Target points	IC_50_ or EC_50_(μmol/L)		References
				SARS-CoV-2 3CLpro,	SARS-CoV	
Glycyrrhetinic acid	*Glycyrrhizae Radix*	Anti-inflammatory and anti-viral	Inhibits SARS virus replication	534.6	–	[57, 86, 87]
Cepharanthine	Stephania Japonica	Anti-viral, anti-infection	Inhibit the mutation of the novel coronavirus. Inhibits S protein binding to ACE2 via calcium channels, upregulates intracellular cholesterol levels and inhibits viral infection	–	0.417	[88, 89]
Matrine	*Radix Sophorae Flavescentis*	Antiviral, anti-inflammatory, immunomodulatory	Acts on TNF-α, IL-6 and CASP3 targets in the TNF signaling pathway to regulate viral replication, apoptosis and inflammatory responses	–	–	[90]
Safflower yellow pigment A	Saffron	Anti-inflammatory, analgesic	Reduction of inflammatory factors by decreasing the level of IL-1β and TNF-α	–	–	[91]
Baicalin	*Scutellaria baicalensis*	Anti-inflammatory	Inhibits the expression of IL-2, IL-6 and TNF-α, inhibits NF-κB mRNA expression and phosphorylation of p38	0.39	–	[29, 92]
Naringenin	Chinese Ephedra	Reduction of lung inflammation	Inhibits the release of inflammatory mediators through the NF-κB pathway, inhibits lung neutrophil infiltration and TNF-α secretion, and attenuates neutrophil-mediated oxidative damage	–	–	[27]
Quercetin	Forsythia, honeysuckle, ginkgo	Anti-inflammatory and antioxidant	Reduction of the expression level of NF-κB and ICAM-1 to weaken the role of NF-κB signaling pathway to reduce the inflammatory response. And it prevents viral spikes from binding to the body by inhibiting ACE2	12.65	–	[86, 93–96]
Lignocaine	Perrin	Anti-inflammatory, antibacterial, antiviral	Inhibits viral replication by inhibition of preprotein convertase and influence of viral replication in vitro by inhibition of expression of the capsid protein I complex	74.86	–	[41, 94–96]
Kaempferol	*Fructus Forsythiae*, Lonicera Japonica	Anti-inflammatory, antioxidant, antiviral	Inhibits the expression of TNF-α, IL-6, IL-10, IL-1β to down-regulate the activity of MAKP, NF-κB and other inflammatory pathways	21.7	–	[93, 96]
β-sitosterol	*Fructus Forsythiae*, Lonicera Japonica	Hypolipidemic, anti-inflammatory, anti-allergic	Inhibits TNF-α- NF-κB and TβR1-Smad2/3 signaling pathways ameliorate liver fibrosis injury	115		[97, 98]
Tectorigenin	*Polygonum Cuspidatum*	Anti-viral	Has high affinity to SARS-CoV-2 3CLpro, SARS-CoV 3CLpro and MERS-CoV 3CLpro, can effectively inhibit viral replication is a potential broad-spectrum inhibitor of coronavirus	18.66	–	[62, 99]
Artesunate	*Artemisia annua*	Inhibition of inflammatory response	Reduces the expression levels of IL-6, MCP-1 and TNF-α	–	–	[100]
Forsythiaside A	*Fructus Forsythiae*	Anti-viral	Inhibits SARS-CoV-2 3CLpro in vitro	3.18		[23]
Honokiol	Thicket	Anti-inflammatory, antioxidant	Inhibits JKN and NF-κB mediated inflammatory factor signaling pathways, reduces oxidative stress, and exerts anti-inflammatory effects	–	233.4	[29]
Resveratrol	*Polygonum Cuspidatum,* Grape	Anti-inflammatory, anti-viral	Inhibits TNF-α and NF-κB expression to reduce the expression of pro-inflammatory factors	29.81	–	[99, 101]

## Conclusion and the future

China has a considerable natural advantage in the application of natural products, and the fact that Professor Tu Youyou has been awarded the 2015 Nobel Prize in Physiology or Medicine for the discovery of artemisinin has proved the recognition of the world to the research on natural products of our country. The world is also highly concerned about natural products, which have a wide range of applications in drugs, cosmetics and health products, etc. The several coronavirus pandemics since the twenty-first century remind us that we should look for solutions to prevent and treat coronavirus as soon as possible. Natural products usually treat diseases through multiple targets and pathways with few side effects, which can compensate for many disadvantages of chemical drug treatments. At present, many natural product related drugs have been used clinically, with potent results in the combination of Western and Chinese medicine, and the prospects for their use and development remain vast. Similar to China, India, the United States and many European countries have increased investment in natural products for the treatment of coronavirus-related diseases, and the world is concerned about the future of natural products.

Natural products have a broad development potential. In the early stages of the COVID-19 outbreak, the antiviral activity and clinical efficacy of Redesivir and chloroquine, which were expected to be high, were not satisfactory [102]. Natural products make up a larger share of drug development, and many, such as taxol, have become established clinical agents. Natural molecule compounds are multi-pathway and multi-targeted, with potential broad-spectrum antibacterial and antiviral activity. Many clinical trials have also demonstrated the efficacy of natural molecule compounds formulations in combination with basic drugs in the treatment of COVID-19 patients. However, compared to chemical drugs, there is less research data and clinical trials related to natural drugs, and the safety and efficacy of natural products have not been demonstrated in detail. The active ingredients of some plants have therapeutic potential, but there is no sound scientific evidence to support it. We should use modern scientific methods to analyse the pharmacological activity of natural products in detail, so as to provide reliable scientific basis for the research and development of new drugs.

In the development of natural products, there is also a need for research direction. The active ingredients of natural products are related to the origin and the extraction process. For the genuine medicinal materials, mass production should be carried out without destroying the active ingredients, and endangered plants should be protected. In addition, the extraction process of active ingredients may affect the effectiveness of active ingredients. For original plants with multiple active ingredients, appropriate processes should be developed according to the characteristics of the extracted compounds to maximize the activity of the extracts.

For more mature natural products, relevent clinical trials should be promoted. Clinical study cases should be expanded, groups should be refined, multi-center randomised controlled trials and combination treatment protocols should be added, their clinical safety and efficacy should be evaluated in detail, and effective treatment protocols should be included in treatment guidelines so that disease treatment can be based on evidence. This is of great significance for the promotion of natural products.

## Data Availability

The datasets presented in this review can be found in online repositories.

## Abbreviations

COVID-19: Coronavirus disease 2019
WHO: World Health Organization
CET: Central European time
BST: Beijing standard time
NCCP: Novel coronavirus pneumonia patient
RNA: Ribose nucleic acid
SARS: Severe acute respiratory syndrome
MERS: Middle east respiratory syndrome
ACE2: Angiotensin-converting enzyme II
HIV: Human immunodeficiency virus
FDA: Food and drug administration
USA: The Unite State of America
MODS: Multiple organ dysfunction syndrome
SARS-CoV-2: Severe acute respiratory syndrome coronavirus 2
NF-κB: Nuclear factor kappa-B
IL-1: Interleukin-1
IL-6: Interleukin-6
TNF-α: Tumor necrosis factor-α
IκB: Inhibitor of NF-κB
TCR: T cell receptor
JAK1: Janus kinase 1
STAT3: Signal transducer and activator of transcription 3
MMP-9: Matrix metalloprotein-9
TIMP1: Tissue inhibitors of metalloproteinase 1
DC: Dendritic cell
LPS: Lipopolysaccharide
PSI: Pneumonia severity index
influenza A H1N1: Influenza A hemagglutinin 1 neuraminidase 1
MCP-1: Monocyte chemotactic protein 1
MAPK3: Mitogen-activated protein kinase 3
PTGS: Post-transcriptional gene silencing
IP-10: Interferon-inducible protein-10
3CLpro: 3C-likeprotease
M pro: Main protease
MERS-CoV: Middle East respiratory syndrome coronavirus
HCV: Hepatitis C virus
